# Genetically diverse mice are novel and valuable models of age-associated susceptibility to *Mycobacterium tuberculosis*

**DOI:** 10.1186/s12979-014-0024-6

**Published:** 2014-12-16

**Authors:** David E Harrison, Clinton M Astle, M Khalid Khan Niazi, Samuel Major, Gillian L Beamer

**Affiliations:** The Jackson Laboratory, 600 Main Street, Bar Harbor, ME 04609 USA; The Ohio State University, Columbus, OH 43210 USA; Tufts University Cummings School of Veterinary Medicine, 200 Westboro Road, Grafton, MA 01536 USA

**Keywords:** Tuberculosis, Rapamycin, HET3, DO, Diversity outbred, Genetically diverse population, Early Secreted Antigenic Target-6 (ESAT-6)

## Abstract

**Background:**

Tuberculosis, the disease due to *Mycobacterium tuberculosis*, is an important cause of morbidity and mortality in the elderly. Use of mouse models may accelerate insight into the disease and tests of therapies since mice age thirty times faster than humans. However, the majority of TB research relies on inbred mouse strains, and these results might not extrapolate well to the genetically diverse human population. We report here the first tests of *M. tuberculosis* infection in genetically heterogeneous aging mice, testing if old mice benefit from rapamycin.

**Findings:**

We find that genetically diverse aging mice are much more susceptible than young mice to *M. tuberculosis,* as are aging human beings. We also find that rapamycin boosts immune responses during primary infection but fails to increase survival.

**Conclusions:**

Genetically diverse mouse models provide a valuable resource to study how age influences responses and susceptibility to pathogens and to test interventions. Additionally, surrogate markers such as immune measures may not predict whether interventions improve survival.

## Findings

### Introduction

The importance of using genetically diverse experimental animals to model human populations was demonstrated many years ago in aging research [1] to avoid confusing a single genetic individual’s unique characteristics with those of the species. Here, we use two models of genetically diverse mice to study susceptibility to *M. tuberculosis* infection and immune responses to an *M. tuberculosis* antigen, Early Secreted Antigenic Target-6 (ESAT-6), in old mice.

The first population is HET3 mice, the F2 progeny from crossing CByB6F1/J F1 hybrid females (JAX stock number 100009, [BALB/cByJ females and C57BL/6J males]) with C3D2F1/J F1 hybrid males (JAX stock number 100004, [C3H/HeJ females and DBA/2J males]). As defined by Roderick [2], the resultant “four way cross” population is reproducible, and each HET3 mouse is genetically unique but a full sibling of all other mice in the population. We used HET3 mice in studies of primary *M. tuberculosis* infection and aging and in a rapamycin intervention.

The second population is Diversity Outbred (DO) mice (JAX stock number 009376). DO mice are derived from the eight parental inbred strains (A/J, C57BL/6J, 129S1/SvImJ, NOD/ShiLtJ, NZO/HILtJ, CAST/EiJ, PWK/PhJ, and WSB/EiJ) [3,4], and thus are more genetically diverse than HET3 mice. We used aging DO mice to test immune responses to ESAT-6 in the context of rapamycin intervention.

In humans, age-related declines in T cell function [5] increase risk of developing tuberculosis (TB) due to reactivation of a latent infection or following primary infection with *M. tuberculosis* [6,7]. T cell functions are also defective in aging mice infected with *M. tuberculosis* [8-10], making mouse models useful for testing if boosting immunity through pharmaceuticals or vaccination might help protect the elderly against this deadly disease.

We tested whether rapamycin treatment alters acquired immunity to primary *M. tuberculosis* infection and to vaccination with the *M. tuberculosis* protein antigen ESAT-6 plus an adjuvant. We chose rapamycin because it has multiple potential benefits for aging and infectious disease models. Studies in the Harrison laboratory show rapamycin improves acquired cellular and humoral immunity in old mice but not young mice (unpublished). Additionally, rapamycin also extends lifespan [11-13] and controls energy/nutrient utilization of immune cells [14]. Furthermore, Jagganath et al. [15] demonstrated that rapamycin co-administered with *M. bovis* BCG-vaccination enhanced *M. tuberculosis* antigen-specific interferon-gamma (IFN-γ) responses and reduced *M. tuberculosis* load in young, C57BL/6 mice. However, genetically diverse mice, aged mice, and survival were not tested. Here, rapamycin boosted antigen-specific IFN-γ in genetically diverse aged mice with primary *M. tuberculosis* infection (HET3 mice) and following vaccination (DO mice), but did not alter survival to *M. tuberculosis* primary infection. This shows the importance of survival measures, which is highly relevant to *M. tuberculosis* studies because correlates of protective immunity to infection or to vaccination are not known [16-18].

## Methods

### Ethics statement

Experiments were approved by The Jackson Laboratory IACUC protocols (DEH02-01; DEH07-08; DEH95-01) and Tufts University IACUC protocols (G2012-53; G2012-151). For both institutions, approved criteria for euthanasia due to morbidity included unresponsiveness, labored breathing, sunken hips, difficulty walking, cachexia, or persistent social isolation. Biosafety Level 3 work was approved by Tufts University Institutional Biosafety Committee registration (GRIA04).

### Mice and rapamycin administration

Mice were bred, aged in-house until 22 months of age, and treated with rapamycin at The Jackson Laboratory (Bar Harbor, ME) as detailed [19]. Treatment was for 6 weeks followed by one month withdrawal; control mice received chow alone. Rapamycin treatment on this schedule enhances multiple aspects of immunity in old but not young mice (unpublished, DH).

### Infection with *Mycobacterium tuberculosis*

Female HET3 mice were shipped to the New England Regional Biosafety Laboratory (Grafton MA) and acclimated during the rapamycin withdrawal period. Fifty-one aging (24.5 months) and 18 young (6 months) mice received 94 ± 30 *M. tuberculosis* Erdman bacilli in the lungs using a CH Technologies® (Westwood, NJ) machine. Mice were monitored daily. Prior to infection, mice were weighed weekly. After infection, mice were weighed at least twice per week. Data from 11 aging HET3 mice were censored due to neoplasia (N = 8), hydrouterus (n = 2), or sudden death with no gross lesions (N = 1). Thus, 40 aging HET3 mice gave useful data.

### Vaccination with recombinant ESAT-6

Fifty-six female DO mice were fed rapamycin or control diet as above, shipped to the Cummings School of Veterinary Medicine (Grafton, MA) and acclimated during the rapamycin withdrawal period. At 24.5 months of age, 36 DO mice (18 rapamycin diet at 14 ppm; 18 control diet) were vaccinated three times, two weeks apart, subcutaneously with ESAT-6 or adjuvant as described [20]. Nine DO mice (5 rapamycin at 14 ppm; 4 control diet) received the adjuvant alone.

### Whole blood interferon gamma release assays

Blood was collected from the submandibular vein of HET3 mice prior to infection and monthly throughout. Blood was obtained from the heart of DO mice following euthanasia. ESAT-6 specific IFN-γ was quantified by ELISA or by ELIPSOT [21] except that blood was diluted 1:5, not 1:10.

### Flow cytometry

Blood was collected from the submandibular vein 6 weeks prior to *M. tuberculosis* infection of HET 3 mice; red blood cells were lysed and nucleated cells were counted. Forward and side scatter profiles were adjusted to eliminate debris and isotype controls used for gating. Naïve CD4 T cells were defined as CD45.2^+^CD3^+^CD4^+^CD62L^hi^CD44^lo^ using the following antibodies: CD45.2 FITC clone 104.7; CD3ε PE clone 145-2C11; CD4 Pacific Orange clone GK1.5; CD62L PE-Cy7 clone Mel-14; CD44 APC-Cy7 clone IM7.8. Samples were read using a four-laser/13-color BD LSRII special order system (340551) analytical cytometer (BDBiosciences, San Jose, CA) and analyzed by FlowJo software at the Flow Cytometry Laboratory of The Jackson Laboratory (Bar Harbor, ME). In DO mice, ESAT-6 specific CD4 T cell proliferation was assessed by intranuclear incorporation of BrdU following manufacturer instructions (BrdU Flow Kit, BD Biosciences). Samples were read and analyzed as described [21] except an AccuriC6 flow cytometer was used.

### Histology

Formalin-inflated lungs were fixed, processed, embedded in paraffin, cut at 5 μm, and stained with hematoxylin and eosin at the Cummings School of Veterinary Medicine Histology Laboratory. Two serial sections, 100 μm apart, were examined by a board certified veterinary pathologist (GB) without knowledge of the groups.

### Statistical analyses

**Figure 1:**
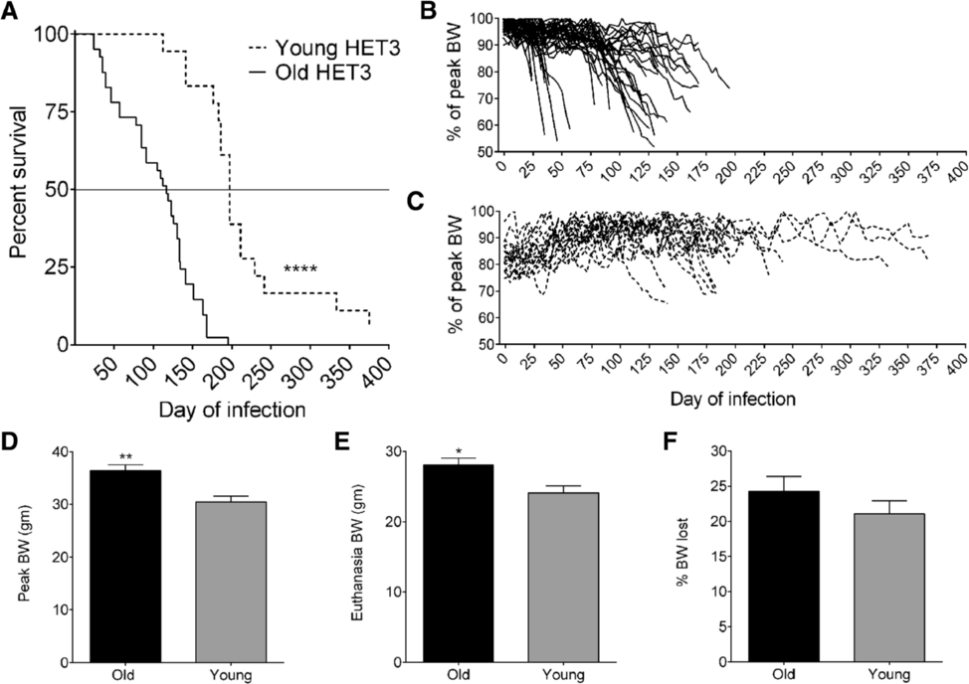
**Aged HET3 mice are more susceptible to** ***M. tuberculosis*** **than young.** *M. tuberculosis* infected old (N = 40) and young (N = 18), female HET3 mice were euthanized when morbidity developed. Survival **(A)**, weights over time in old **(B)** and young **(C)** mice, peak body weight **(D)**, weight at euthanasia **(E)**, and proportional weight loss **(F)** are shown. Survival data were analyzed by Log rank test, ****p < 0.0001. Pairwise data were analyzed by Student’s *t-*tests, *p < 0.05, **p < 0.01. Data were censored for 11 old mice due to non-TB morbidity. No data from young mice were censored.

Using GraphPad Prism 6.4, data were analyzed for outliers by ROUT and Grubb’s methods. No outliers were identified by ROUT. Grubb’s identified one outlier at 444 SFU. This value was excluded because it was not clear whether it reflected technical error or a true biological effect. Data were then analyzed for the distribution. Data were not normally distributed so Spearman correlation coefficients were calculated. For Figure 1B, exponential and 4^th^ degree polynomial regression analyses were performed; the decay of the rate of weight loss resulted in an exponential model, written as f(x) = 0.5749 * e ^(−0.0122*x)^ with x representing the time in days. The polynomial model (4^th^ degree) is not reported due to its tendency to overfit. Survival curves were analyzed by Logrank analysis. Multigroup comparisons were analyzed using one-way ANOVA with Tukey’s post-test. Significance for all tests was defined *p < 0.05, **p < 0.01, ***p < 0.001, and ****p < 0.0001.

## Results and discussion

### Genetically diverse aging mice are more susceptible to *Mycobacterium tuberculosis* than young

Mouse models of genetic diversity are becoming more widely used [4,13,22-25]. A majority of *M. tuberculosis* research in young and old mice has used the C57BL/6 inbred strain, with fewer studies using B6 hybrids or other inbred strains such as Balb/c, C3H/HeJ, DBA/2, CBA/J, I/St, and A/Sn [26]. Through these studies we have learned valuable information regarding responses to *M. tuberculosis,* characteristics of susceptible and resistant strains, and genes that contribute to susceptibility. However, the allelic homozygosity of inbred strains leads to expression of deleterious recessive genes, which may affect results especially in aging studies (1). Therefore, genetically diverse mouse models may be useful for *M. tuberculosis* research.

As with human beings, aging genetically diverse HET3 mice are more susceptible to aerosolized *M. tuberculosis* than young, with median survivals of 118 and 197 days in aging and young mice, respectively (Figure 1A). A characteristic of TB in mice and human beings is weight loss; in fact, weight is a useful indicator of TB in mice because it occurs before respiratory, motor, and social disturbances [27]. And because weighing mice is non-invasive, repeated measures can be performed on individual mice with little stress. In aging HET3 mice, weight loss began earlier in *M. tuberculosis* infection than in young mice (Figure 1B,C), even though aging mice were heavier (Figure 1D,E). Aging mice also lost relatively more weight due to TB (Figure 1F).

Evidence of clinical disease began on average, 21 days after *M. tuberculosis* infection in old mice; in contrast disease in young mice began, on average, 127 days after infection. This likely reflects better control of *M. tuberculosis* bacilli, or better control of detrimental inflammation, in young mice. The duration of disease was the same in aging and young HET3 mice (85 ± 50 versus 88 ± 41 days, respectively). Regardless of age, lungs eventually fill with inflammatory cells and variable necrosis in all mice (not shown). Therefore, the main effect of old age appears to be that disease onset occurs earlier.

**Figure 2:**
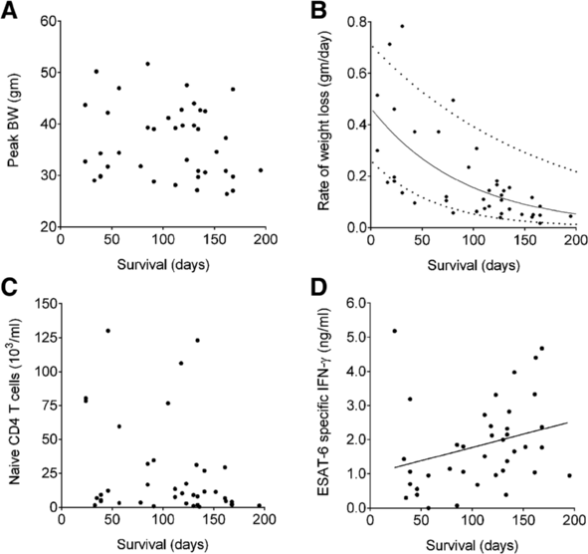
**Body weight, immune parameters, and survival in aging** ***M. tuberculosis*** **infected HET3 mice.** Peak body weights of *M. tuberculosis* infected aging mice (N = 40) **(A)** and the rate of weight loss **(B)** as compared to survival. There was no significant correlation between peak body weight and survival (Spearman r −0.1082, p = 0.26) **(A)**. A strong negative correlation was identified between the rate of weight loss and survival (Spearman r −0.7123, p < 0.0001) with dashed lines showing the 95% confidence intervals **(B)**. Blood was sampled to quantify naïve CD4 T cells prior to infection and then after infection at monthly intervals to quantify the average ESAT-6-specific IFN-γ responses for each mouse. No significant correlation was identified between naïve CD4 T cells and survival (Spearman r −0.2775, p < 0.08) **(C)**, but there was a weak positive, statistically significant correlation between ESAT-6 specific IFN-γ and survival (Spearman r 0.3694, p < 0.02) **(D)**. Data were censored for 11 old mice due to non-TB morbidity.

We expected that body weight would positively correlate with survival. However, this was not true for aging HET3 mice (Figure 2A), but we did observe that the rate of weight loss was the best indicator of TB disease progression, shown and modeled by an exponential decay (Figure 2B). Although weight changes do not reflect specific immunologic or pathologic changes, the ability to track body weight provides a foundation to identify biomarkers that precede weight loss.

We explored relationships between survival and immunity that may be important for resistance to *M. tuberculosis*. Survival did not correlate with the total (not shown) or naïve CD4 T cell numbers prior to infection (Figure 2C). Survival correlated significantly with ESAT-6 specific IFN-γ in HET3 mice but the correlation was weak (Figure 2D) and some mice were clear exceptions.

In summary, our findings suggest that aged, genetically diverse mice can model age-related susceptibility to *M. tuberculosis* primary infection in humans. Although T cell responses are important for control of *M. tuberculosis* (shown by markedly increased susceptibility when these molecules are absent in inbred mice [28,29]), in immune competent aged HET3 mice, antigen-specific IFN-γ is a positive but weak correlate of survival.

### Rapamycin boosts antigen-specific interferon-gamma responses

**Figure 3:**
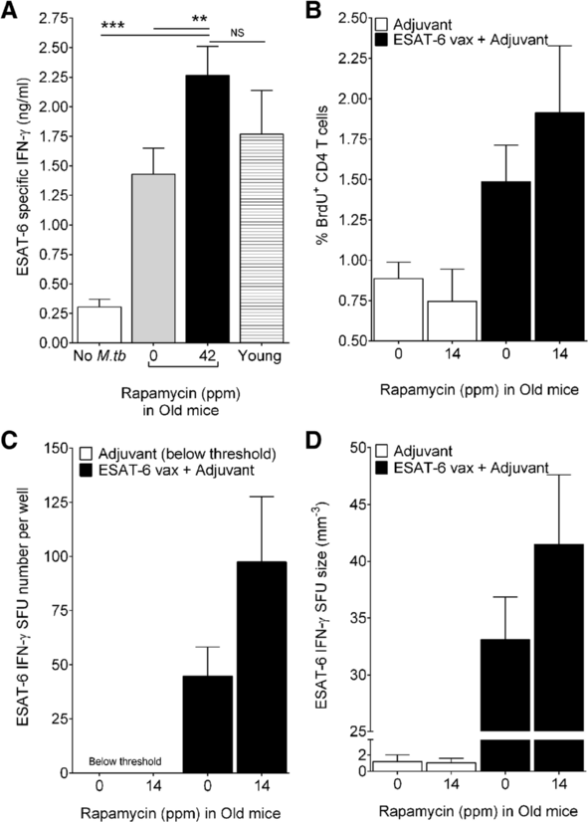
**Rapamycin enhances ESAT-6 responses to primary** ***M. tuberculosis*** **infection and to ESAT-6 vaccination.** Rapamycin-treated and untreated old HET3 mice were infected with *M. tuberculosis* by aerosol. Old DO mice were vaccinated with ESAT-6 plus adjuvant or adjuvant alone. ESAT-6 specific IFN-γ responses the blood of HET3 mice were quantified before and after infection **(A)**. Three weeks after the final vaccination of DO mice, proliferating ESAT-6 specific CD4 T cells **(B)** and cells capable of ESAT-6 specific IFN-γ **(C, D)** were determined by ELISPOT. Results are from 12–20 mice per group, reported as average + SEM, analyzed by one-way ANOVA, **p < 0.01, ***p < 0.001, NS not significant.

Interventions to improve the length and quality of life are of interest, and HET3 mice are advantageous for research and testing interventions because the population of siblings is reproducible and results can be compared across time and from different laboratories [13,19,30]. Furthermore, rapamycin benefits multiple types of immune responses in old mice but not young mice (unpublished, DH), and rapamycin could improve outcome to *M. tuberculosis* infection by enhancing innate or acquired immunity. Rapamycin has beneficial effects on lifespan [11-13] and controls metabolism of immune cells [14] important for resistance to *M. tuberculosis* [28,29]. Rapamycin also stimulates *M. tuberculosis* antigen-specific TH1 cells [15,31] by inducing autophagy [32] which can eliminate intracellular *M. tuberculosis* bacilli [33]. We thus tested whether rapamycin enhanced immune responses to *M. tuberculosis* in aging mice. Indeed, rapamycin increased ESAT-6-specific IFN-γ responses during *M. tuberculosis* infection (Figure 3A). Rapamycin also increased ESAT-6-specific proliferation, the numbers of IFN-γ producing cells, and amount of IFN-γ produced by the responding cells (Figure 3B-D) when ESAT-6 was administered as a vaccine. Whether these responses to ESAT-6 vaccination improve control of *M. tuberculosis* bacillary growth or prolong survival remains to be determined.

In summary, our results indicate that oral rapamycin showed trends for enhancing ESAT-6 specific responses in genetically diverse old mice. The variable statistical significance likely reflects the relatively small sample size, in particular with the vaccination studies in DO mice. Although additional studies are needed, the finding may be relevant for elderly people because oral delivery of agents is an attractive method to improve immune function.

### Rapamycin does not improve survival of aging mice with primary *M.tb* infection

**Figure 4:**
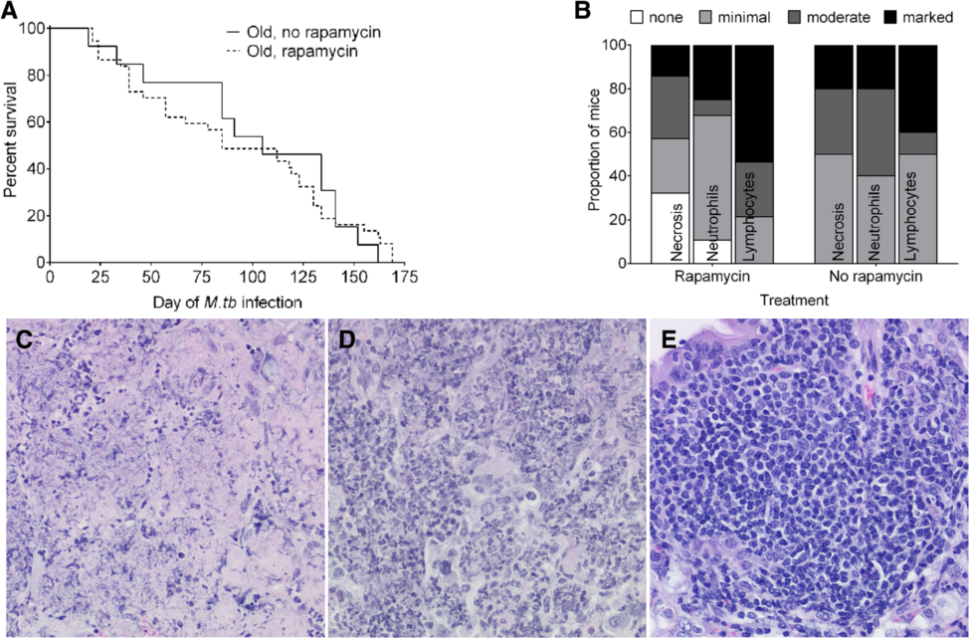
**Oral rapamycin does not improve survival of** ***M. tuberculosis*** **infected aged mice and minimally impacts lung lesions.** Rapamycin treated (N = 37) and untreated (N = 13) old HET3 mice were infected with aerosolized *M. tuberculosis* and euthanized when removal criteria were met **(A)**. Lung sections were semi-quantitatively evaluated for necrosis, neutrophils, and lymphocytes, and the proportion of mice was determined for each category **(B)**. Examples of the microscopic lesions are shown, magnified 200 times: necrosis **(C)**; neutrophils **(D)**; lymphocytes **(E)**.

We next assessed whether rapamycin treatment improved survival, delayed the onset of TB disease, or altered the lung lesions during primary *M. tuberculosis* infection. Rapamycin did not prolong survival or extend the median survival (Figure 4A) or delay disease onset (not shown). Modest changes in lungs are attributable to rapamycin: a slight reduction in the proportion of mice with marked necrosis and neutrophil influx and a shift toward increased lymphocytes (Figure 4B). Regardless, lung damage in all mice was substantial, and thus, despite having potential benefits (on lifespan, cellular and humoral immunity, autophagy), rapamycin in our model did not delay TB disease or extend survival in *M. tuberculosis* infected aged HET3 mice regardless of immunologic changes or tissue architectural changes.

We observed a trend for rapamycin-enhanced antigen-specific responses (proliferation, IFN-γ) in the vaccination model. It is unknown whether rapamycin or the enhanced immune responses can actually protect against *M. tuberculosis* challenge in this model, but this is a logical next step for future studies. However, clinical outcomes and survival measures in mice are now even more important when testing interventional or preventative therapies against *M. tuberculosis* infection because protective correlates of immunity are not fully known [16-18]. This strategy is necessary to assess the potential of rapamycin to improve vaccination efficacy in elderly people.

## Abbreviations

BW: Body weight
ESAT-6: Early secreted antigenic target-6
*M.tb*: *Mycobacterium tuberculosis*
SFU: Spot forming units
TB: Tuberculosis
